# Thermodynamic coupling between folding correctors and the first of dimerized nucleotide binding domains in CFTR

**DOI:** 10.21203/rs.3.rs-6890276/v1

**Published:** 2025-06-17

**Authors:** Guangyu Wang

**Affiliations:** 1 Department of Physiology and Membrane Biology, University of California School of Medicine, Davis, CA, USA; 2 Department of Drug Research and Development, Institute of Biophysical Medico-chemistry, Reno, NV, USA

**Keywords:** allosteric pathway, cooperative folding, digital biology, energy landscape, melting threshold, noncovalent thermoring structure, protective folding, protein stability

## Abstract

The most common cystic fibrosis mutation is the F508del mutation in the human cystic fibrosis transmembrane conductance regulator (hCFTR), which causes misfolding of the first of two nucleotide binding domains (NBD1/2), preventing Mg/ATP-dependent NBD dimerization for normal function. Although folding correctors elexacaftor/VX-445 and lumacaftor/VX-809 have been combined to correct the NBD1 misfolding, the exact correction pathway is still unknown. In this study, the constrained tertiary noncovalent interaction networks or the thermoring structures of dimerized NBD1 in hCFTR/E1371Q with or without F508del were analyzed to identify the weakest noncovalent bridge as the final posttranslational tertiary folding of dimerized NBD1 in response to folding correctors. These computational analyses suggested that hCFTR may primarily use cooperative folding between α- and β-subdomains in dimerized NBD1 as the last step upon the binding of the potentiator ivacaftor/VX-770. However, the binding of folding correctors may allosterically protect the α-subdomain from misfolding until subsequent core formation. This thermodynamic protective mechanism, unlike the chaperone-based one in cotranslational NBD1 folding, may restore posttranslational NBD1 folding for tight Mg/ATP-mediated NBD dimerization in the F508del mutation, and also potentially apply to treating other cystic fibrosis patients with rare mutations.

## INTRODUCTION

Protecting group strategy is typically used in multi-step organic syntheses to control the length and efficiency of the desired reactivity (1). Similarly, proteins also employ several protective strategies such as chaperones, quality control mechanisms, and other factors during the folding process to ensure correct 3D structure and to prevent misfolding or aggregation (2–3). A good example is the cotranslational folding of the first of two cytosolic nucleotide-binding domains (NBD1/2) from the cystic fibrosis transmembrane conductance regulator (CFTR) among ATP-binding cassette (ABC) transporters.

The CFTR channel is a multi-domain polytopic protein located in the apical membrane of epithelial cells, regulating ion and fluid homeostasis in various tissues (4). The NBD1/2 interface intracellular loops (ICL1–4) that extend from two 6-spanning transmembrane domains (TMD2/1) in a domain-swapping manner. A relatively unstructured regulatory (R) domain inserts between NBD1/2 and TMD1/2 (5). When the R domain is released upon phosphorylation by protein kinase A (PKA), Mg/ATP-mediated NBD1-NBD2 dimerization rearranges TMD1-TMD2 interactions, leading to channel opening. This opening is stabilized by the hydrolysis-deficient E1371Q mutation (6–9).

NBD1 consists of N-terminal (residues 389–491), α-helical (residues 500–564), and parallel-four-stranded β-sheet core (residues 568–603) subdomains (10). These subdomains undergo sequential synthesis, starting with ATP-stimulated N-terminal compaction (11), and ending with C-terminal compaction (3). The timing of these folding events is tightly controlled to ensure that α-helical subdomain collapse is delayed until the β-sheet core is synthesized. To prevent misfolding, it is necessary to protect the α-subdomain, possibly with chaperones in the ribosome during synthesis to achieve efficient cotranslational folding without any off-pathway products (12).

On the other hand, considering that the most common cystic fibrosis mutation F508del damages CFTR folding by destabilizing NBD1 or its interactions with ICL4, leading to a failure of Mg/ATP-dependent NBD dimerization for normal channel gating (13–28), the systematic fluidic grid-like noncovalent interaction mesh networks of NBD1, with or without the interactions with ICL4, have been constrained as thermoring structures with the minimum energy required to stabilize the interactions (29–30). Although the biggest thermoring Grid_18_ of the isolated (F508del)hNBD1 monomer is located in the N-terminal subdomain, and has a calculated melting temperature threshold (T_m,th_) of 32 °C to unfold the weakest N396-N445 H-bond (29), the biggest thermoring Grid_14_ of the partially dimerized NBD1 of full-length (F508del)hCFTR with elexacaftor/VX445 bound is positioned in the α-subdomain and has a calculated T_m,th_ of 39 °C to unfold the weakest D529-R555 salt bridge. Furthermore, in the tightly dimerized NBD1 of full-length (F508del)hCFTR with Trikafta (ivacaftor/VX-770, tezacaftor/VX-661 and elexacaftor/VX445) bound, the biggest Grid_10_ is also present in the α-subdomain and has a calculated T_m,th_ of 49 °C to unfold the weakest Y517-D537 H-bond (30).

Since the weakest noncovalent interaction for the final posttranslational folding in NBD1 is located in the region that accumulates numerous suppressor mutations, which improve NBD1 stability, CFTR folding efficiency, and (F508del)hCFTR processing with folding correctors such as VX-445 or VX-809 synergistically (22–24, 31–35), it is of special interest to explore the hypothesis that the binding of folding correctors initiates a protective posttranslational pathway to prevent NBD1 misfolding in (F508del)hCFTR in the absence of chaperones.

To test this hypothesis, the thermoring structures of dimerized NBD1 of hCFTR with or without F508del were further analyzed in the presence of single or combined modulators. The results demonstrated that the weakest Y517-D537 or D529-R555 H-bond appeared in the α-subdomain of dimerized NBD1 from hCFTR with or without F508del upon the binding of single or combined folding correctors such as VX445, VX-661 or VX-809. In contrast, the least-stable Q525-E585 H-bond linked both α and β-subdomains in hCFTR with the potentiator ivacaftor/VX-770 bound. Therefore, dimerized NBD1 in full-length hCFTR may employ a cooperative pathway in the final posttranslational folding. However, the binding of the folding corrector to hCFTR may stimulate a protective pathway in dimerized NBD1, which was used to correct the structural defect of (F508del)hCFTR.

## COMPUTATIONAL METHODS

### Data mining resources

Four cryo-EM structures of phosphorylated and Mg/ATP-bound hCFTR/E1371Q constructs in an activated state at 4 °C were selected for thermoring analysis. Three structures with F508 included 6O2P with VX-770 bound (model resolution = 3.3 Å) (36), 7SV7 with VX-661 bound (model resolution = 3.8 Å) (28), and 7SVD with VX-809 bound (model resolution = 2.7 Å) (28). One structure without F508 was 8EIO with VX445/VX-809 bound (model resolution = 2.8 Å) (28).

### Standard methods for filtering non-covalent interactions

The standard methods for filtering non-covalent interactions, along with exact calculations, were the same as those previously used, ensuring accurate and repeatable results (29–30, 37–43). UCSF Chimera was used to review potential stereo-selective or regio-selective intra-domain lateral noncovalent interactions along the single polypeptide chain of hNBD1 with or without F508. These interactions included salt bridges, H-bonds and lone pair/CH/cation-π interactions between paired amino acid side chains. When a backbone NH or CO at a residue position was within a cutoff distance for an H-bond with a side chain of a nearby residue, that H-bond was also considered. Detailed cutoff distances and interaction angles can be obtained in the online Supporting Information (Tables S1, S2, S3, and S4). Notably, momentary fluctuation-induced perturbations in noncovalent interactions during protein dynamics were not taken into account. In this study, approximately 36–43 different noncovalent interactions were identified along the single polypeptide chain from L383/E384 to L636/Q637 of NBD1 on each protomer.

### Mapping the energy landscape of tertiary folding of NBD1 using the grid thermodynamic model

The same grid thermodynamic model that was previously validated was used to map the energy landscape of tertiary folding of NBD1 in full-length hCFTR with or without F508 (29–30, 37–43). Briefly, a black line represented the specific polypeptide chain from the N-terminal to the C-terminal of NBD1 while colorful lines represented specific noncovalent interactions linked by side chains of paired protein residues (colorful arrows). When these lines formed a systematic fluidic grid-like mesh network, each noncovalent interaction had at least two paths between paired residues. A direct path was the interaction itself while a reverse path consisted of the nearby peptide segment and other noncovalent interactions. Since the reverse path could be shortened by Floyd-Warshall’s Algorithm as the minimal free or silent residues that did not involve any noncovalent interactions (44), the shortest direct and reverse pathways created a thermo-sensitive ring or thermoring, denoted as Grids, where “s” represented the size of a thermoring or the total number of free or silent residues to control the weakest noncovalent interaction within it. For example, when E391 formed an H-bond with K447 in Figure 2a, if a direct path started from E391 and ended at K447, the peptide segment from K447 to E391 paved a reverse path. Since another adjacent reverse path from K447 to F446, M394, and E391 had a length of 2 (the total number of free amino acids in peptide segments K447 to F446 and 394 to 391), and no other possible adjacent reverse paths were shorter than 2, the shortest round path from E391 to M394, F446, K447 and back to E391 lined a thermoring Grid_2_ with a 2-residue size to control the least-stable E391-K447 H-bond.

Once each noncovalent interaction was assigned a unique size, the biggest thermoring could be identified to trace the weakest noncovalent interaction along the single peptide chain. Meanwhile, the total noncovalent interactions and grid sizes along the same polypeptide chain could be summed up and displayed by black and cyan circles next to the mesh network map.

### Calculation of the melting temperature threshold (T_m,th_) of NBD1

The same empirical equation and coefficients used in previous studies on temperature-dependent structures were applied to calculate the melting temperature threshold (T_m,th_) of NBD1 (29–30, 37–43):

(1) Tm,thCo=34+n-2×10+20-s×2

where, n represents the total number of basic H-bonds (each approximately 1 kcal/mol) that are calculated to be approximately equal in stability to the least-stable or weakest noncovalent interaction controlled by the biggest grid (45); and s is the grid size used to control the least-stable noncovalent interaction in the biggest grid.

### Evaluation of the grid-based systemic thermal instability (T_i_) of NBD1

The same empirical equation used in previous studies on temperature-dependent structures was utilized to calculate the systematic thermal instability (T_i_) of NBD1 (29–30, 37–43):

(2) $${\text{T}}_{\text{i}}=\text{S}/\text{N}$$

where, S and N are the total grid sizes and the total non-covalent interactions along a specific polypeptide chain of NBD1, respectively. This calculation allows for evaluation of NBD1’s compact conformational entropy or flexibility.

## RESULTS

### Biggest thermoring of dimerized (F508del)hNBD1 is located in the α-subdomain upon VX-445/VX-809 binding to TMD1/2

The specific binding of VX-809 and VX-445 to TMD1 and the TMD2/lasso interface is sufficient to restore normal Mg/ATP-mediated NBD1-NBD2 dimerization of (F508del)hCFTR/E1371Q without the potentiator VX-770 (28). Therefore, it is intriguing to explore the impacts of folding correctors on the location and stability of the biggest thermoring in (F508del)hNBD1.

**Figure 1.**
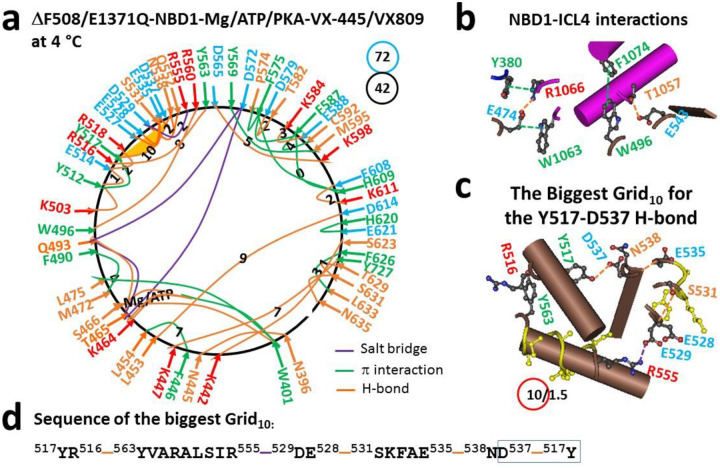
The thermoring structures of phosphorylated hCFTR/E1371Q/ΔF508 with VX-445 and VX-809 bound in the activated state at 4 °C. (**a**) The grid-like noncovalently interacting mesh network based on the cryo-EM structure of hCFTR/E1371Q/ΔF508 with VX-445 and VX-809 bound in the presence of Mg/ATP/PKA at 4 °C (PDB ID, 8EIO, 2.8 Å). Salt bridges, H-bonds and π interactions are colored purple, orange, and green, respectively. The constrained grid sizes required to control the least-stable noncovalent interactions in the grids are labeled with black numbers. The least-stable Y517-D537 H-bond in the biggest Grid_10_ is highlighted. The total grid sizes and the total grid size-controlled noncovalent interactions along the single peptide chain of NBD1 from L383 to L636 are shown in cyan and black circles, respectively. (**b**) Noncovalent interactions at the NBD1/ICL4 interface (Y380 is at the TMD1-NBD1 linker). (**c**) The structure of the biggest Grid_10_ with a 10-residue size to control the least-stable Y517-D537 H-bond. The grid size and the equivalent basic H-bonds for the least-stable noncovalent interaction are shown in and near a red circle. (**d**) The sequence of the biggest Grid_10_ to control the least-stable Y517-D537 H-bond in the blue box.

The dimerized (F508del)hNBD1 (PDB, 8EIO) is a single polypeptide spanning from L383 to L636 with a disordered regulatory insertion (RI) (residues E403 to L436) (28). Along this peptide chain, forty-two intra-domain non-covalent interactions via amino acid side chains were located in the N-terminal, α- and β-core subdomains (Figure 1a). Along with tight Mg^2+^ binding to T465, Q493 and D572 and tight ATP binding to W401, K464, T465, S466 and Q493, the Q552-D529-R555 bridges formed a smaller Grid_2_ in the α-subdomain while F575, F587 and H609 produced the smallest Grid_0_ in the β-subdomain (Figure 1a). Collectively, a total of 72 grid sizes and a total of 42 noncovalent interactions were calculated as their ratio for a systematic thermal stability (T_i_) of 1.71 (Table 1). Therefore, dimerized hNBD1 was tightly folded. Meanwhile, hNBD1 also interacted with ICL4 via E474-R1066 and E543-T1057 H-bonds and several π interactions such as Y380-R1066, E474-W1063, and W496-F1074 (Figure 1b).

On the other hand, despite the tight coupling between the N- and C-termini via K442-S623-K447 and L453-D614 backbone H-bonds, the biggest Grid_10_ was found in the α-subdomain along with weakened interfacial interactions between NBD1 and ICL4. With a 10-residue size controlling the least-stable Y517-D537 H-bond which was equivalent to 1.5 basic H-bonds, the calculated melting threshold (T_m,th_) for the thermoring from Y517, E528, S531, D537, and back to Y517 was about 49 °C (Table 1), higher than the T_m,th_ of 39 °C for (F508del)hNBD1 with VX-445 bound (30). This result was consistent with the notion that the corrector VX-445 fails to confer enough NBD1 stability to poorly responsive variants (32).

If the location of the biggest Grid_10_ in the α-subdomain is a result of weakened ICL4-NBD1 interactions in (F508del)hCFTR rather than the binding of VX445 and VX-809 to TMD1/2, then the binding of folding correctors to hCFTR/E1371Q should reposition the biggest thermoring in NBD1. To investigate this, the thermoring structures of NBD1 in hCFTR/E1371Q were analyzed in response to different modulators.

### Biggest thermoring of dimerized hNBD1 involves both α- and β-subdomains upon VX-770 binding to the TMD1/TMD2 interface

**Figure 2.**
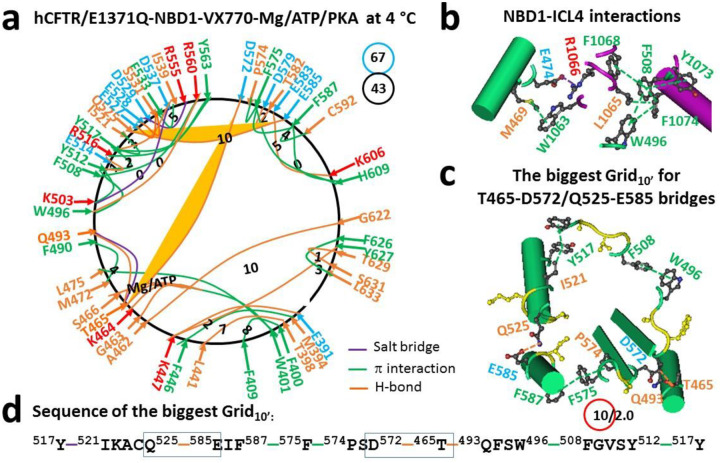
The thermoring structures of phosphorylated hCFTR/E1371Q with VX-770 bound in the activated state at 4 °C. (**a**) The grid-like noncovalently interacting mesh network based on the cryo-EM structure of hCFTR/E1371Q in the presence of Mg/ATP/PKA and VX-770 at 4 °C (PDB ID, 6O2P, 3.3 Å). Salt bridges, H-bonds and π interactions are colored purple, orange, and green, respectively. The constrained grid sizes required to control the least-stable noncovalent interactions in the grids are labeled with black numbers. The least-stable T465-D572 and Q525-E585 H-bonds in the biggest Grid_10’_ is highlighted. The total grid sizes and the total grid size-controlled noncovalent interactions along the single peptide chain of NBD1 from E384 to Q637 are shown in cyan and black circles, respectively. (**b**) Noncovalent interactions at the NBD1/ICL4 interface. (**c**) The structure of the biggest Grid_10’_ with a 10-residue size to control the least-stable T465-D572 and Q525-E585 H-bonds. The grid size and the equivalent basic H-bonds for the least-stable noncovalent interaction are shown in and near a red circle. (**d**) The sequence of the biggest Grid_10’_ to control the least-stable T465-D572 and Q525-E585 H-bonds in the blue boxes.

After the potentiator VX-770 bound to the TMD1/TMD2 interface of hCFTR/E1371Q, forty-three intra-domain non-covalent interactions via amino acid side chains were identified in dimerized NBD1 (Figure 2a). NBD1 is a single polypeptide from L383 to Q637 and includes the disordered RI from E410 to L436 (36). When compared with (F508del)hCFTR/E1371Q with VX-445 and VX809 bound (PDB, 8EIO), although the ICL4-NBD1 interactions were enhanced by the additional M469-W1063 and F1068/Y1073-F508-F1074-L1065 π interactions (Figure 2b), and ATP still tightly bound to W401, K464, T465, S466 and Q493, along with the additional E391-K447 and T398-L441 H-bonds and the supplemental M394-F446 and F400-F409 π interactions, the metal bridges between D572 and T465 or Q493 were disrupted (Figure 2a). As a result, several changes were observed in α- and β-subdomains (Figure 2a). For example, when the D529-Q552 H-bond was replaced with the R516-Y563-Y517 π interactions in the α-subdomain, the Y569-M595 π interaction, as well as the Y565-K598, K584-E588, E608-K611 and S631-N635 H-bonds were disrupted in the β-subdomain. In this case, when the K503-Y512-Y517-D537-K503 noncovalent bridges formed the new smallest Grid_0_ in the α-subdomain, Q525 H-bonded with E585 via their side chains, coupling both α- and β-subdomains. Meanwhile, the T465-D572, A462-G622 and K447-Y627 H-bonds still linked N- and C-termini together (Figure 2a). However, the total grid sizes decreased from 72 to 67. Therefore, the systematic thermal instability (T_i_) decreased from 1.71 to 1.56.

Notably, the biggest Grid_10’_ linked both α- and β-subdomains via a thermoring from Y517 to I521, Q525, E585, F587, F575, P574, D572, T465, Q493, W496, F508, Y512, and back to Y517 (Figure 2c–d). When the Q525-E585 and T465-D572 H-bonds were energetically equivalent to 2.0 basic H-bonds (2.0 kcal/mol), the calculated T_m,th_ was about 54 °C (Table 1), which was higher than the T_m,th_ of 50 °C in NBD1 of hCFTR/E1371Q (PDB, 6MSM) (30). Therefore, although ivacaftor/VX-770 destabilizes full-length F508del-CFTR and accelerates channel deactivation at 37 °C (46), it actually potentiates hCFTR activity by stabilizing cooperative folding between the α- and β-subdomains (30).

### Biggest thermoring of dimerized hNBD1 remains in the α-subdomain upon VX-661 binding to TMD1

**Figure 3.**
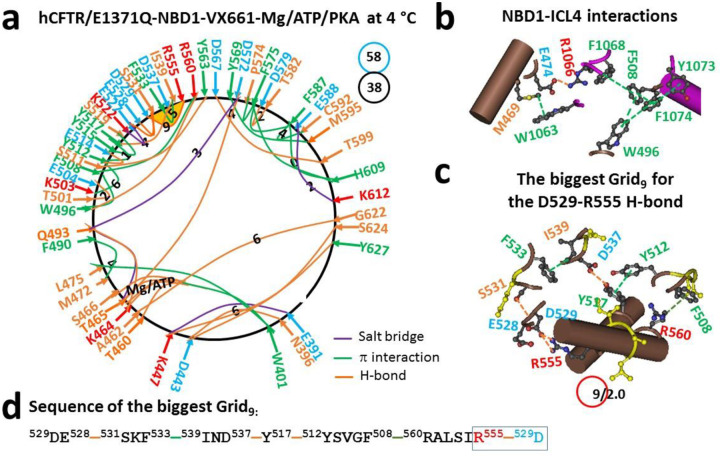
The thermoring structures of phosphorylated hCFTR/E1371Q with VX-661 bound in the activated state at 4 °C. (**a**) The grid-like noncovalently interacting mesh network based on the cryo-EM structure of hCFTR/E1371Q in the presence of Mg/ATP/PKA and VX-661 at 4 °C (PDB ID, 7SV7, 3.8 Å). Salt bridges, H-bonds and π interactions are colored purple, orange, and green, respectively. The constrained grid sizes required to control the least-stable noncovalent interactions in the grids are labeled with black numbers. The least-stable D529-R555 H-bond in the biggest Grid_9_ is highlighted. The total grid sizes and the total grid size-controlled noncovalent interactions along the single peptide chain of NBD1 from E384 to Q637 are shown in cyan and black circles, respectively. (**b**) Noncovalent interactions at the NBD1/ICL4 interface. (**c**) The structure of the biggest Grid_9_ with a 9-residue size to control the least-stable D529-R555 H-bond. The grid size and the equivalent basic H-bonds for the least-stable noncovalent interaction are shown in and near a red circle. (**d**) The sequence of the biggest Grid_9_ to control the least-stable D529-R555 H-bond in the blue box.

When VX-770 was replaced with VX-661, the different binding site in TMD1 disrupted the F508-Y1073 and L1065-F1074 π interactions at the NBD1-ICL4 interface, inducing a global change in NBD1 despite intact ATP binding (Figure 3a–b) (28, 36). In the N-terminal subdomain, the M394-F446 and F400-F409 π interactions and the T398-L441 H-bond were substituted by the N396-D443 H-bond. In the α subdomain, the K503-D537 salt bridge, as well as the R516/Y517-Y563 π interactions were disrupted. Meanwhile, the K522-E527 salt bridge, the T501-E504 H-bond, and the Y515-S519 π interaction were created. Following the disruption of the Q525-E585 H-bond between α and β subdomains, the E583-K606 and T529-S531 H-bonds, together with the F626-L633 π interaction were replaced with a new D567-T599 H-bond and the E588-K612 salt bridge. Taken as a whole, a total of noncovalent interactions and grid sizes decreased from 43 to 38 and from 67 to 58, respectively. Therefore, the systematic thermal instability (T_i_) also decreased from 1.56 to 1.53 (Table 1). Although the T465-D572, A462-G622, K447-Y627 and D443-S624 H-bonds still coupled the N-terminal with the β-core subdomain, the biggest Grid_9_ was identified in the α subdomain to control the D529-R555 H-bond via a thermoring from D529, E528, S531, F533, I539, D537, Y517, Y512, F508, R560, R555, and back to D529 (Figure 3c–d). When the controlled H-bond was energetically equivalent to 2 basic H-bonds, the calculated T_m,th_ was about 56 °C (Table 1), which was higher than the T_m,th_ of 50 °C of NBD1 in hCFTR/E1371Q (PDB, 6MSM) (30). Therefore, VX-661 binding to TMD1 relocated the final folding in the α-subdomain and enhanced the stability of NBD1, regardless of the change in the ICL4-NBD1 interactions.

### Biggest thermoring of dimerized NBD1 also remains in the α-subdomain upon VX-809 binding to TMD1

**Figure 4.**
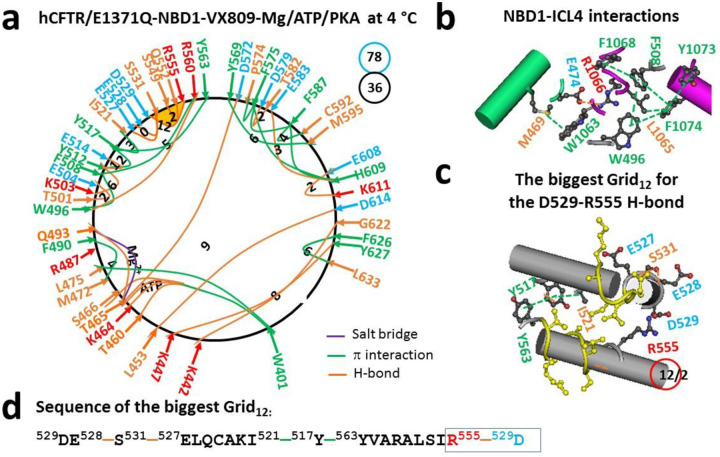
The thermoring structures of phosphorylated hCFTR/E1371Q with VX-809 bound in the activated state at 4 °C. (**a**) The grid-like noncovalently interacting mesh network based on the cryo-EM structure of hCFTR/E1371Q in the presence of Mg/ATP/PKA and VX-809 at 4 °C (PDB ID, 7SVD, 2.7 Å). Salt bridges, H-bonds and π interactions are colored purple, orange, and green, respectively. The constrained grid sizes required to control the least-stable noncovalent interactions in the grids are labeled with black numbers. The least-stable D529-R555 H-bond in the biggest Grid_12_ is highlighted. The total grid sizes and the total grid size-controlled noncovalent interactions along the single peptide chain of NBD1 from E384 to Q637 are shown in cyan and black circles, respectively. (**b**) Noncovalent interactions at the NBD1/ICL4 interface. (**c**) The structure of the biggest Grid_12_ with a 12-residue size to control the least-stable D529-R555 H-bond. The grid size and the equivalent basic H-bonds for the least-stable noncovalent interaction are shown in and near a red circle. (**d**) The sequence of the biggest Grid_12_ to control the least-stable D529-R555 H-bond in the blue box.

VX809 and VX661 share the same binding site (28), leading to similar effects on the tertiary structure of NBD1 and the NBD1-ICL4 interface (Figure 4a–b). Restoring the L1065-F1074 π interaction at the interface disrupted the weakest Q525-E585 H-bond between the α- and β-subdomains. As a result, in the α-subdomain, the K522-E527 salt bridge, the Y517-D537 H-bond and the Y515-S519 and F533-I539 π interactions were replaced by the S549-Q552 H-bond and the Y517-Y563 π interaction. Meanwhile, in the β-core subdomain, an H-bond shifted from D567-T599 to E583-H609 along with replacing the E588-K612 salt bridge with the E608-K611 H-bond. However, the T629-S631 H-bond in the C-terminus disappeared. Lastly, while the H-bonds shifted from A462-G622 and D443-S624 to L453-D614 and K442-S623, respectively, at the interface between the N- and C-termini, the E391-K447 salt bridge and the N396-D443 H-bond in the N-terminal disappeared but the F626-L633 π interaction appeared in the C-terminal. Overall, the total grid sizes changed from 58 to 76 along with a decrease in the total noncovalent interactions from 38 to 36 (Figure 4a), resulting in an increase in systematic thermal instability (T_i_) from 1.53 to 2.11 (Table 1).

On the other hand, the weakest D529-R555 H-bond was influenced by the biggest Grid_12_ in the α-subdomain. Hence, the calculated T_m,th_ was about 50 °C, which was the same as the T_m,th_ of 50 °C for NBD1 in hCFTR/E1371Q (PDB, 6MSM) (30). In this context, the binding of VX-809 to TMD1 also caused a relocation of the final folding in the α-subdomain, regardless of the change in the NBD1-ICL4 interactions.

## DISCUSSION

The folding kinetics of NBD1 in hCFTR are complex and can be influenced by various environmental factors. The NBD1-NBD2 dimerization through bound Mg/ATP is essential for normal CFTR activity, but this process, when disrupted by the misfolding of (F508del)hNBD1, can be restored by the binding of folding correctors like VX-445 and VX-809 to TMD1/2, highlighting the importance of understanding the final step of NBD1 folding for the development of an allosteric correction pathway. In this study, the effects of different modulators on the thermoring structures of dimerized NBD1 with Mg/ATP bound in full-length hCFTR/E1371Q with or without the F508 deletion were examined. The comparative subdomain interactions, T_m,th_ values and drug behaviors suggest that the final step of posttranslational NBD1 folding under physiological condition may involve either a cooperative or protective mechanism, which is lacking in F508-deleted hCFTR. While the cooperative interaction between α and β-subdomains in dimerized NBD1 was still present with the binding of VX-770 to the TMD1/TMD2 interface, the protective step was initiated by the binding of correctors, regardless of the presence of the F508del mutation. Therefore, this protective folding pathway in dimerized NBD1, activated by correctors to rectify the misfolding caused by the F508del mutation, could serve as a potential strategy for developing drugs to treat patients with rare CF mutations in the future.

### The Q525-E585 H-bond promotes cooperative folding coupling between α- and β- subdomains

**Figure 5.**
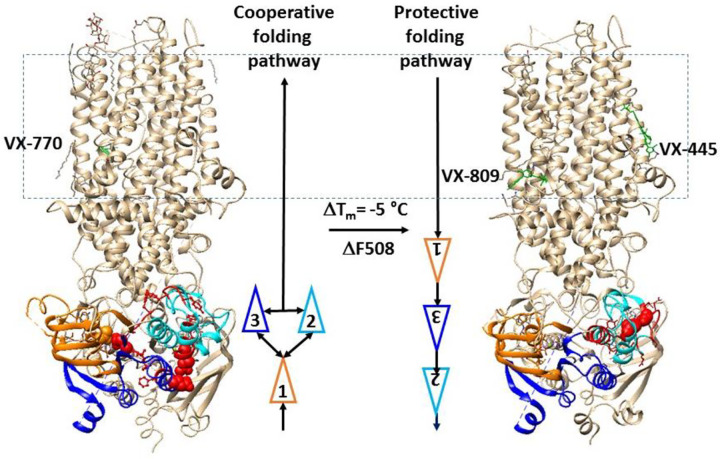
Cooperative and protective folding pathways of NBD1 in hCFTR in response to modulators. Cryo-EM structures of open phosphorylated hCFTR/E1371Q with Mg/ATP/VX770 bound (PDB: 6O2P) and open phosphorylated hCFTR/ΔF508/E1371Q with Mg/ATP/VX809/VX445 bound (PDB: 8EIO) are used for the models. VX770, VX-445 and VX-809 are colored green. The N-terminal, α- and β- subdomains in NBD1 are colored orange, cyan and blue, respectively. The biggest thermorings in NBD1 are shown in red. The residues responsible for the least-stable noncovalent interactions in NBD1 are shown in space fills.

Several lines of evidence have demonstrated that the coupling between ICL4 and NBD1 facilitates NBD1 and full-length CFTR folding (23–24, 35). A recent study further showed that the Q525-E585 H-bond between α- and β-subdomains is present in the isolated hNBD1 monomer but absent upon the F508 deletion. When the regulatory insertion (RI) (residues 400–439) and extension (RE) (residue 645–675) are removed for Mg/ATP-mediated NBD dimerization, it disappears in hNBD1 but appears in (F508del)hNBD1. It was also found in the (F508del)hNBD1 monomer with 3S mutations (29). In contrast, this H-bond appears again in dimerized NBD1 of full-length hCFTR/E1371Q with or without VX-770 bound but disappears in dimerized NBD1 of full-length hCFTR/E1371Q or (F508del)hCFTR/E1371Q with VX-661 or VX-809 or VX-445 bound (Figures 1a, 2a, 3a, 4a) (30). Therefore, although the removal of RI and RE or the introduction of the 3S mutations restores the cooperative folding pathway used in isolated NBD1 from full-length hCFTR, it was compromised in NBD1 of (F508del)hCFTR with folding correctors bound. In this case, an alternative protective folding pathway was needed (Figure 5).

### Protective folding pathway in dimerized (F508del)hNBD1 in response to folding correctors

A previous study revealed that delaying α-subdomain compaction favors cotranslational folding of isolated hNBD1 (3, 12). This study further demonstrated that finalizing α-subdomain compaction also facilitates post-translational folding of dimerized NBD1 in full-length hCFTR with or without F508 in response to folding correctors. Following the binding of a single folding corrector VX-661, VX-809 or VX-445, the weakest D529-R555 salt bridge was always present in the α-subdomain of dimerized or partially dimerized NBD1 (Figures 3a and 4a) (30). Similarly, when two folding correctors VX-445 and VX-809 or VX-661 bind to (F508del)hCFTR, the weakest Y517-D537 H-bond also appeared in the α-subdomain of dimerized NBD1 (Figures 1a) (30). These results suggested that the formation of the weakest D529-R555 or Y517-D537 bridge was the last event in the post-translational folding pathway of dimerized NBD1 in the presence of folding correctors.

Notably, type I correctors have been reported to enhance the stability of (F508del)hCFTR at the plasma membrane at 37 °C (46–48). However, VX-445 does not provide enough NBD1 stability for poorly responsive variants (32), consistent with the lower T_m,th_ of 39 °C of partially dimerized NBD1 in (F508del)hCFTR/E1371Q with VX-445 bound (30). This study further indicated that the combination of VX-445 with VX809 is needed for the higher T_m,th_ of 49 °C to unfold dimerized NBD1 in (F508del)hCFTR/E1371Q (Figure 1c–d, Table 1) (30). Similarly, when Trikafta modulators bind to (F508del)hCFTR, the T_m,th_ needed to unfold dimerized NBD1 also increases to 49 °C (30). Thus, the additional binding of the potentiator VX770 may account for why Trikafta significantly boosts the activity of (F508del)hCFTR more than VX-445 and VX-809 at 37 °C (48).

On the other hand, folding correctors lumacaftor/VX-809 and presumably tezacaftor/VX-661 have been shown to optimize CFTR folding during synthesis in cells (34). This suggests that the folding corrector may act as a chaperone, allosterically regulating the critical D529-R555 or Y517-D537 bridge to ensure the proper cotranslational folding of NBD1. Therefore, the type-I folding correctors VX-809 and VX-661 not only stabilize TMD1 early in biogenesis (49), but also initiate a protective pathway to enhance the efficiency of NBD1 folding from cotranslation to posttranslation. This ultimately corrects structural defects for proper (F508del)hCFTR synthesis and function.

### D529-R555 salt bridge primes correct NBD1 folding

A recent study identified three conserved thermoring anchors in isolated NBD1 monomer or dimer with or without F508 or 3S mutations (29). The first is the smallest Grid_0_ formed by the T465-Mg-Q493 and K464/T465/S466-ATP bridges in the N-terminal subdomain upon Mg/ATP binding. The second is the smaller Grid_2_ shaped by Q552-D529-R555 H-bonds in the α-subdomain. The third is the smaller Grid_3_ lined by E583-K606 and F587-H609 bridges in the β-subdomain. Although these three smaller thermorings are also conserved in dimerized NBD1 of full-length hCFTR/E1371Q (30), the binding of modulators to TMD1 or TMD2 or their interface disrupted the D529-Q552 or E583-K606 H-bond in dimerized NBD1 of hCFTR/E1371Q (Figures 1a, 2a, 3a, 4a) (30). More importantly, the binding of single folding corrector VX-661, VX-809 or VX-445 rendered the D529-R555 salt bridge the weakest in NBD1 no matter whether F508del is introduced or not (Figures 3a & 4a) (30). Therefore, despite complex CFTR NBD1 folding kinetics, the highly-conserved D529-R555 salt bridge is required for normal NBD1 folding upon Mg/ATP binding. In support of this proposal, the D529F or R555K mutation significantly improves NBD1 and full-length CFTR folding (24), possibly by restoring or enhancing kinetic coupling between α-helical subdomain and β-sheet core (Figure 2a), or finalizing the folding of α-helical subdomain (Figures 3a and 4a) (3, 12, 29–30). On the contrary, as the nearby L558S mutation disrupts the coordinated compaction of α-helical and β-sheet core subdomains, which cannot be restored by the introduction of S492P and I539T (PT) (50), this critical D529-R555 salt bridge may be kinetically and thermodynamically impaired. Therefore, no matter whether a cooperative or protective folding pathway is used during CFTR biosynthesis, the highly conserved D529-R555 salt bridge, together with the highly-conserved Mg/ATP binding site, is always needed for correct NBD1 folding in response to complex environment perturbations. Further mutations and molecular dynamic (MD) simulations in this region are required to illuminate the protective role of the salt bridge in NBD1 folding and CFTR trafficking.

## CONCLUSIONS

Noncovalent interactions such as H-bonds, π interactions and salt bridges play a crucial role in protein folding and stability. However, the energy landscape of these interactions is not fully understood. Based on high resolution 3D structures of NBD1 from hCFTR, noncovalent interaction networks were constrained as “thermorings” of various sizes from the smallest to the biggest to control the melting temperature of each noncovalent interaction. This allows for minimal energy requirements to regulate the timing of protein biosynthesis in different scenarios. Although chaperones are typically used to protect a subdomain from cotranslational misfolding in the ribosome, the weakest noncovalent link can also protect a subdomain from posttranslational misfolding. This is essential for proper protein function in response to chemical perturbations. These findings underscore the significance of an intrinsic thermodynamic protection strategy in the post-translational folding of proteins and the management of relevant inherited diseases.

## Supplementary Files

This is a list of supplementary files associated with this preprint. Click to download.

- SupportingInformationdimerizedNBD1andFoldingcorrectorsinhCFTRv1.0.pdf

## Figures and Tables

**Table 1:** Grid thermodynamic model-based new parameters of NBD1 in hCFTR constructs.

Construct	hCFTR/E1371Q			
PDB ID	6O2P	7SV7	7SVD	8EIO
F508	+	-	-	-
Mg/ATP (10 mM)/PKA	+	+	+	+
Bound modulator, VX-	770	661	809	445/809
Sampling temperature, °C	4	4	4	4
NBD dimerized	+	+	+	+
Normal Mg^2+^ site	-	-	-	+
Name of the biggest grid in α-subdomain	Grid_10’_	Grid_9_	Grid_12_	Grid_10_
Involving α/β-subdomain	+	-	-	-
Grid size (s)	10	9	12	10
# of energetically equivalent basic H-bonds (n) controlled by Grid_s_	2.0	2.0	2.0	1.5
Total non-covalent interactions (N)	43	38	36	42
Total grid sizes (S), a.a.	67	58	76	72
Systematic thermal instability (T_i_)	1.56	1.53	2.11	1.71
Calculated T_m,th,_ °C	54	56	50	49

## Data Availability

All data generated or analyzed during this study are included in this published article and Supporting Information.

## ABBREVIATIONS

ABC: ATP-binding cassette
CF: cystic fibrosis
cryo-EM: cryoelectron microscopy
CFTR: cystic fibrosis transmembrane conductance regulator
hCFTR: human CFTR
ICLi: intracellular loop i (i=1,2,3,4)
MD: molecular dynamics
NBD1: nucleotide binding domain 1
NBD2: nucleotide binding domain 2
PKA: protein kinase A
R: regulatory
RE: regulatory extension
RI: regulatory insert
T_i_: systematic thermal instability
T_m,th_: melting temperature threshold
TMD1: transmembrane domain 1
TMD2: transmembrane domain 2

## References

[1] Schelhaas M. Waldmann H. Protecting Group Strategies in Organic Synthesis. Angew. Chem.Int Ed in Engl. (1996). 35: 2056–2083. 10.1002/anie.199620561

[2] Díaz-Villanueva JF, Díaz-Molina R, García-González V. Protein Folding and Mechanisms of Proteostasis. Int J Mol Sci. 2015 Jul 28;16(8):17193–230. doi: 10.3390/ijms160817193. 26225966 PMC4581189

[3] McDonald EF, Meiler J, Plate L. CFTR Folding: From Structure and Proteostasis to Cystic Fibrosis Personalized Medicine. ACS Chem Biol. 2023 Oct 20;18(10):2128–2143. doi: 10.1021/acschembio.3c00310. 37730207 PMC10595991

[4] Cutting GR. Cystic fibrosis genetics: from molecular understanding to clinical application. Nat Rev Genet. 2015 Jan;16(1):45–56. doi: 10.1038/nrg3849. 25404111 PMC4364438

[5] Liu F, Zhang Z, Csanády L, Gadsby DC, Chen J. Molecular Structure of the Human CFTR Ion Channel. Cell. 2017 Mar 23;169(1):85–95.e8. doi: 10.1016/j.cell.2017.02.024. 28340353

[6] Cheng SH, Rich DP, Marshall J, Gregory RJ, Welsh MJ, Smith AE. Phosphorylation of the R domain by cAMP-dependent protein kinase regulates the CFTR chloride channel. Cell. 1991 Sep 6;66(5):1027–36. doi: 10.1016/0092-8674(91)90446-6. 1716180

[7] Vergani P, Lockless SW, Nairn AC, Gadsby DC. CFTR channel opening by ATP-driven tight dimerization of its nucleotide-binding domains. Nature. 2005 Feb 24;433(7028):876–80. doi: 10.1038/nature03313. 15729345 PMC2756053

[8] Zhang Z, Liu F, Chen J. Conformational Changes of CFTR upon Phosphorylation and ATP Binding. Cell. 2017 Jul 27;170(3):483–491.e8. doi: 10.1016/j.cell.2017.06.041. 28735752

[9] Zhang Z, Liu F, Chen J. Molecular structure of the ATP-bound, phosphorylated human CFTR. Proc Natl Acad Sci U S A. 2018 Dec 11;115(50):12757–12762. doi: 10.1073/pnas.1815287115. 30459277 PMC6294961

[10] Lewis HA, Buchanan SG, Burley SK, Conners K, Dickey M, Dorwart M, Fowler R, Gao X, Guggino WB, Hendrickson WA, Hunt JF, Kearins MC, Lorimer D, Maloney PC, Post KW, Rajashankar KR, Rutter ME, Sauder JM, Shriver S, Thibodeau PH, Thomas PJ, Zhang M, Zhao X, Emtage S. Structure of nucleotide-binding domain 1 of the cystic fibrosis transmembrane conductance regulator. EMBO J. 2004 Jan 28;23(2):282–93. doi: 10.1038/sj.emboj.7600040. 14685259 PMC1271750

[11] Khushoo A, Yang Z, Johnson AE, Skach WR. Ligand-driven vectorial folding of ribosome-bound human CFTR NBD1. Mol Cell. 2011 Mar 18;41(6):682–92. doi: 10.1016/j.molcel.2011.02.027. 21419343 PMC3095512

[12] Kim SJ, Yoon JS, Shishido H, Yang Z, Rooney LA, Barral JM, Skach WR. Translational tuning optimizes nascent protein folding in cells. Science. 2015 Apr 24;348(6233):444–8. 25908822 10.1126/science.aaa3974

[13] Cheng SH, Gregory RJ, Marshall J, Paul S, Souza DW, White GA, O’Riordan CR, Smith AE. Defective intracellular transport and processing of CFTR is the molecular basis of most cystic fibrosis. Cell. 1990 Nov 16;63(4):827–34. doi: 10.1016/0092-8674(90)90148-8. 1699669

[14] Lukacs GL, Chang XB, Bear C, Kartner N, Mohamed A, Riordan JR, Grinstein S. The delta F508 mutation decreases the stability of cystic fibrosis transmembrane conductance regulator in the plasma membrane. Determination of functional half-lives on transfected cells. J Biol Chem. 1993 Oct 15;268(29):21592–8. 7691813

[15] Qu BH, Strickland EH, Thomas PJ. Localization and suppression of a kinetic defect in cystic fibrosis transmembrane conductance regulator folding. J Biol Chem. 1997 Jun 20;272(25):15739–44. doi: 10.1074/jbc.272.25.15739. 9188468

[16] Sharma M, Benharouga M, Hu W, Lukacs GL. Conformational and temperature-sensitive stability defects of the delta F508 cystic fibrosis transmembrane conductance regulator in post-endoplasmic reticulum compartments. J Biol Chem. 2001 Mar 23;276(12):8942–50. doi: 10.1074/jbc.M009172200. 11124952

[17] Du K, Sharma M, Lukacs GL. The DeltaF508 cystic fibrosis mutation impairs domain-domain interactions and arrests post-translational folding of CFTR. Nat Struct Mol Biol. 2005 Jan;12(1):17–25. doi: 10.1038/nsmb882. 15619635

[18] Wang C, Protasevich I, Yang Z, Seehausen D, Skalak T, Zhao X, Atwell S, Spencer Emtage J, Wetmore DR, Brouillette CG, Hunt JF. Integrated biophysical studies implicate partial unfolding of NBD1 of CFTR in the molecular pathogenesis of F508del cystic fibrosis. Protein Sci. 2010 Oct;19(10):1932–47. doi: 10.1002/pro.480. 20687163 PMC2998727

[19] Protasevich I, Yang Z, Wang C, Atwell S, Zhao X, Emtage S, Wetmore D, Hunt JF, Brouillette CG. Thermal unfolding studies show the disease causing F508del mutation in CFTR thermodynamically destabilizes nucleotide-binding domain 1. Protein Sci. 2010 Oct;19(10):1917–31. doi: 10.1002/pro.479. 20687133 PMC2998726

[20] Thibodeau PH, Richardson JM 3rd, Wang W, Millen L, Watson J, Mendoza JL, Du K, Fischman S, Senderowitz H, Lukacs GL, Kirk K, Thomas PJ. The cystic fibrosis-causing mutation deltaF508 affects multiple steps in cystic fibrosis transmembrane conductance regulator biogenesis. J Biol Chem. 2010 Nov 12;285(46):35825–35. doi: 10.1074/jbc.M110.131623. 20667826 PMC2975206

[21] Jih KY, Li M, Hwang TC, Bompadre SG. The most common cystic fibrosis-associated mutation destabilizes the dimeric state of the nucleotide-binding domains of CFTR. J Physiol. 2011 Jun 1;589(Pt 11):2719–31. doi: 10.1113/jphysiol.2010.202861. 21486785 PMC3112550

[22] Aleksandrov AA, Kota P, Cui L, Jensen T, Alekseev AE, Reyes S, He L, Gentzsch M, Aleksandrov LA, Dokholyan NV, Riordan JR. Allosteric modulation balances thermodynamic stability and restores function of ΔF508 CFTR. J Mol Biol. 2012 May 25;419(1–2):41–60. doi: 10.1016/j.jmb.2012.03.001. 22406676 PMC3891843

[23] Rabeh WM, Bossard F, Xu H, Okiyoneda T, Bagdany M, Mulvihill CM, Du K, di Bernardo S, Liu Y, Konermann L, Roldan A, Lukacs GL. Correction of both NBD1 energetics and domain interface is required to restore ΔF508 CFTR folding and function. Cell. 2012 Jan 20;148(1–2):150–63. doi: 10.1016/j.cell.2011.11.024. 22265408 PMC3431169

[24] Mendoza JL, Schmidt A, Li Q, Nuvaga E, Barrett T, Bridges RJ, Feranchak AP, Brautigam CA, Thomas PJ. Requirements for efficient correction of ΔF508 CFTR revealed by analyses of evolved sequences. Cell. 2012 Jan 20;148(1–2):164–74. 22265409 10.1016/j.cell.2011.11.023 PMC3266553

[25] He L, Aleksandrov AA, An J, Cui L, Yang Z, Brouillette CG, Riordan JR. Restoration of NBD1 thermal stability is necessary and sufficient to correct ΔF508 CFTR folding and assembly. J Mol Biol. 2015 Jan 16;427(1):106–20. doi: 10.1016/j.jmb.2014.07.026. 25083918 PMC4757845

[26] Chong P. A.; Farber P. J.; Vernon R. M.; Hudson R. P.; Mittermaier A. K.; Forman-Kay J. D. Deletion of Phenylalanine 508 in the First Nucleotide-Binding Domain of the Cystic Fibrosis Transmembrane Conductance Regulator Increases Conformational Exchange and Inhibits Dimerization. J. Biol. Chem. 2015, 290 (38), 22862–22878. 26149808 10.1074/jbc.M115.641134 PMC4645629

[27] Froux L, Coraux C, Sage E, Becq F. Short-term consequences of F508del-CFTR thermal instability on CFTR-dependent transepithelial currents in human airway epithelial cells. Sci Rep. 2019 Sep 24;9(1):13729. doi: 10.1038/s41598-019-50066-7. 31551433 PMC6760155

[28] Fiedorczuk K, Chen J. Molecular structures reveal synergistic rescue of Δ508 CFTR by Trikafta modulators. Science. 2022 Oct 21;378(6617):284–290. doi: 10.1126/science.ade2216. 36264792 PMC9912939

[29] Wang G. ATP-dependent thermoring basis for the heat unfolding of the first nucleotide-binding domain isolated from human CFTR. Res Sq [Preprint]. 2024 Nov 21:rs.3.rs-5479740. doi: 10.21203/rs.3.rs-5479740/v1. Nat Sci. 2025. 5(1–2):e70007. https://doi.org/10.1002/ntls.70007

[30] Wang G. Trikafta rescues F508del-CFTR by tightening specific phosphorylation-dependent interdomain interactions. bioRxiv [Preprint]. 2025 Apr 3:2024.11.20.624197. doi: 10.1101/2024.11.20.624197. Nat Sci. 2025. 5(3):e70009. https://doi.org/10.1002/ntls.70009.

[31] Teem JL, Berger HA, Ostedgaard LS, Rich DP, Tsui LC, Welsh MJ. Identification of revertants for the cystic fibrosis delta F508 mutation using STE6-CFTR chimeras in yeast. Cell. 1993 Apr 23;73(2):335–46. doi: 10.1016/0092-8674(93)90233-g. 7682896

[32] McDonald EF, Kim M, Olson JA 3rd, Meiler J, Plate L. Proteostasis Landscapes of Cystic Fibrosis Variants Reveals Drug Response Vulnerability. bioRxiv [Preprint]. 2025 Jan 17:2024.07.10.602964. doi: 10.1101/2024.07.10.602964. Update in: Proc Natl Acad Sci U S A. 2025 Apr 29;122(17):e2418407122. doi: 10.1073/pnas.2418407122. PMC12054793 40261935

[33] Farinha CM, King-Underwood J, Sousa M, Correia AR, Henriques BJ, Roxo-Rosa M, Da Paula AC, Williams J, Hirst S, Gomes CM, Amaral MD. Revertants, low temperature, and correctors reveal the mechanism of F508del-CFTR rescue by VX-809 and suggest multiple agents for full correction. Chem Biol. 2013 Jul 25;20(7):943–55. doi: 10.1016/j.chembiol.2013.06.004. 23890012

[34] Okiyoneda T, Veit G, Dekkers JF, Bagdany M, Soya N, Xu H, Roldan A, Verkman AS, Kurth M, Simon A, Hegedus T, Beekman JM, Lukacs GL. Mechanism-based corrector combination restores ΔF508-CFTR folding and function. Nat Chem Biol. 2013 Jul;9(7):444–54. doi: 10.1038/nchembio.1253. 23666117 PMC3840170

[35] Soya N, Xu H, Roldan A, Yang Z, Ye H, Jiang F, Premchandar A, Veit G, Cole SPC, Kappes J, Hegedüs T, Lukacs GL. Folding correctors can restore CFTR posttranslational folding landscape by allosteric domain-domain coupling. Nat Commun. 2023 Oct 27;14(1):6868. doi: 10.1038/s41467-023-42586-8. 37891162 PMC10611759

[36] Liu F, Zhang Z, Levit A, Levring J, Touhara KK, Shoichet BK, Chen J. Structural identification of a hotspot on CFTR for potentiation. Science. 2019 Jun 21;364(6446):1184–1188. 31221859 10.1126/science.aaw7611 PMC7184887

[37] Wang G. The Network Basis for the Structural Thermostability and the Functional Thermoactivity of Aldolase B. Molecules. 2023 Feb 15;28(4):1850. doi: 10.3390/molecules28041850. 36838836 PMC9959246

[38] Wang G. Network Basis for the Heat-Adapted Structural Thermostability of Bacterial Class II Fructose Bisphosphate Aldolase. ACS Omega. 2023 May 11;8(20):17731–17739. doi: 10.1021/acsomega.3c00473. 37251155 PMC10210171

[39] Wang G. Thermal Ring-Based Heat Switches in Hyperthermophilic Class II Bacterial Fructose Aldolase. ACS Omega. 2023 Jun 27;8(27):24624–24634. doi: 10.1021/acsomega.3c03001. 37457467 PMC10339327

[40] Wang G. Thermoring-based heat activation switches in the TRPV1 biothermometer. Int J Biol Macromol. 2023 Sep 1;248:125915. doi: 10.1016/j.ijbiomac.2023.125915. 37481175

[41] Wang G. Thermoring basis for the TRPV3 bio-thermometer. Sci Rep. 2023 Dec 7;13(1):21594. doi: 10.1038/s41598-023-47100-0. Erratum in: Sci Rep. 2024 Feb 28;14(1):4909. doi: 10.1038/s41598-024-55395-w. 38062125 PMC10703924

[42] Wang G. Phosphatidylinositol-4,5-biphosphate (PIP2)-Dependent Thermoring Basis for Cold-Sensing of the Transient Receptor Potential Melastatin-8 (TRPM8) Biothermometer. Physchem 2024, 4(2), 106–119; 10.3390/physchem4020008

[43] Wang G. Thermo-ring basis for heat unfolding-induced inactivation in TRPV1. Res Sq [Preprint]. 2024 May 9:rs.3.rs-3280283. doi: 10.21203/rs.3.rs-3280283/v1. Nat. Sci.4, 2024.e20240008. https://doi.org/10.1002/ntls.20240008.

[44] Floyd R. W. Algorithm-97 - Shortest path. Commun Acm. 1962. 5, 345–345.

[45] Neel AJ, Hilton MJ, Sigman MS, Toste FD. Exploiting non-covalent π interactions for catalyst design. Nature. 2017 Mar 29;543(7647):637–646. doi: 10.1038/nature21701. 28358089 PMC5907483

[46] Meng X., Wang Y., Wang X., Wrennall J.A., Rimington T.L., Li H., Cai Z., Ford R.C., and Sheppard D.N. (2017). Two Small Molecules Restore Stability to a Subpopulation of the Cystic Fibrosis Transmembrane Conductance Regulator with the Predominant Disease-causing Mutation. J. Biol. Chem. 292, 3706–3719. 10.1074/jbc.M116.751537. 28087700 PMC5339754

[47] Eckford PD, Ramjeesingh M, Molinski S, Pasyk S, Dekkers JF, Li C, Ahmadi S, Ip W, Chung TE, Du K, Yeger H, Beekman J, Gonska T, Bear CE. VX-809 and related corrector compounds exhibit secondary activity stabilizing active F508del-CFTR after its partial rescue to the cell surface. Chem Biol. 2014 May 22;21(5):666–78. doi: 10.1016/j.chembiol.2014.02.021. 24726831

[48] Veit G, Roldan A, Hancock MA, Da Fonte DF, Xu H, Hussein M, Frenkiel S, Matouk E, Velkov T, Lukacs GL. Allosteric folding correction of F508del and rare CFTR mutants by elexacaftor-tezacaftor-ivacaftor (Trikafta) combination. JCI Insight. 2020 Sep 17;5(18):e139983. doi: 10.1172/jci.insight.139983. 32853178 PMC7526550

[49] Fiedorczuk K, Chen J. Mechanism of CFTR correction by type I folding correctors. Cell. 2022 Jan 6;185(1):158–168.e11. doi: 10.1016/j.cell.2021.12.009. 34995514

[50] Shishido H, Yoon JS, Yang Z, Skach WR. CFTR trafficking mutations disrupt cotranslational protein folding by targeting biosynthetic intermediates. Nat Commun. 2020 Aug 26;11(1):4258. doi: 10.1038/s41467-020-18101-8. 32848127 PMC7450043

